# Influence of aromatase inhibitors therapy on the occurrence of rheumatoid arthritis in women with breast cancer: results from a large population-based study of the Italian Society for Rheumatology

**DOI:** 10.1136/rmdopen-2017-000523

**Published:** 2017-09-28

**Authors:** Marta Caprioli, Greta Carrara, Garifallia Sakellariou, Ettore Silvagni, Carlo Alberto Scirè

**Affiliations:** 1 Division of Rheumatology and Clinical Immunology, Humanitas Clinical and Research Center, Rozzano, Italy; 2 Epidemiology Unit, Italian Society for Rheumatology (SIR), Milan, Italy; 3 Chair and Division of Rheumatology, IRCCS Policlinico San Matteo Foundation, University of Pavia, Pavia, Italy; 4 Rheumatology Unit, Department of Medical Sciences, University of Ferrara, Ferrara, Italy

**Keywords:** tamoxifene, rheumatoid arthritis, aromatase inhibitors, breast cancer

## Abstract

**Objectives:**

The purpose of this study was to evaluate the risk of developing rheumatoid arthritis (RA) in a population of patients with breast cancer treated with aromatase inhibitors (AIs) compared with tamoxifen.

**Methods:**

Data were collected from the administrative healthcare database of Lombardy Region, Italy, from 2004 to 2013. This study follows a nested cohort design, including women with a diagnosis of breast cancer starting treatment with tamoxifen, anastrozole, exemestane or letrozole. The risk of RA related to the prescription of the different drugs was estimated by survival models for competing risks and the results are presented as hazard ratios (HRs) and 95% confidence intervals (95% CI), adjusted for age and cancer severity.

**Results:**

Out of total 10 493 women with breast cancer with a median (IQR) age of 66 (57–74), 7533 (71.8%) started an active treatment with AIs or tamoxifen. In this subgroup a total of 113 new cases of RA developed during the 26 105.9 person-year of 10 186 exposure periods, including time varying exposures in the same patient. Using tamoxifen as reference category, AIs therapy was associated with an increased risk of RA (adjusted HR 1.62 (95%1.03–2.56)), in particular in patients receiving anastrozole, even after adjusting for age and level of neoplasia: (adjusted HR 1.75 (95%1.07–2.86)).

**Conclusions:**

In a large population-based sample of women with breast cancer, exposure to AIs compared with tamoxifen is associated with a significantly increased risk of RA, which is not influenced by the cancer severity and the relationship of age with indication to specific drugs.

**Key messages**

**What is already known about this subject?**

- Some studies suggest a correlation between aromatase inhibitors (AIs) therapy and autoimmune diseases and sporadic case reports have described the onset of rheumatoid arthritis (RA) but whether the exposure to AIs may induce inflammatory arthritis or RA is still an open question.

**What does this study adds?**

- This study has analysed the possible effect of AIs in inducing inflammatory arthritis and RA in exposed patients.
- Our results provide that adjuvant endocrine therapy is associated with a significantly increased risk of developing RA and this is particularly true for anastrozole.

**How might this impact on clinical practice?**

- Occurrence of articular symptoms after exposition to AIs is not only a side effect but may involve a particular pathogenetic pathway which can result in the development of RA.

## Introduction

Adjuvant endocrine therapy is widely used for the treatment of breast cancer and aromatase inhibitors (AIs), such as anastrozole, letrozole and exemestane are the standard first-line treatment in postmenopausal women with tumours positive for estrogen–progesterone receptors.

One of the major side effects of AIs therapy is the development of musculoskeletal symptoms that reduce patient adherence, limiting the evidence-based survival benefits of this class of drugs. Generally, AI-induced musculoskeletal symptoms (AIMSS)^1^ present with symmetrical joint pain often in the wrists and hands.^2^ Carpal tunnel syndrome may be the common cause of pain in these patients as well.^3^

The occurrence of musculoskeletal symptoms in large trials ranges between 5% and 36%,^4^ while studies comparing different AIs have shown no significant difference in the incidence of arthralgia between these treatments.^2^

In several practice-based studies, the incidence rates of AIMSS were higher than those reported in randomised controlled trials (RCTs), ranging from 32% to 47%, with a rate of discontinuation of 13%–50%.^5^

Whether musculoskeletal symptoms can be attributed to an underlying rheumatologic disease remains unknown. Some studies suggest an association between AIs and Sjögren’s syndrome (SS)^6 7^ and a recent study reported two cases of calcinosis, Raynaud phenomenon, oesophageal dysmotility, sclerodactyly, telangiectasia (CREST) syndrome, one of rheumatoid arthritis (RA) and one of SS, with a cumulative incidence of 5.3% of autoimmune disease under AIs treatment.^8^

Sporadic case reports have described the onset of RA in patients who started therapy with AIs^8–10^ or tamoxifen,^11 12^ including the development of multiple subcutaneous rheumatoid nodules on letrozole therapy, improving after treatment discontinuation.^13^

The Clinical, Immunologic and Radiographic Features of the Aromatase Inhibitor Arthralgia Syndrome (CIRAS) study evaluated the association between AIs and clinical and imaging signs of inflammatory arthritis in women with breast cancer with hand pain, showing no significant differences between exposed and unexposed patients in terms of disease activity score, inflammatory markers, diagnosis of RA or other autoimmune disease.^1^ Due to the restriction to patients with pain, the CIRAS study did not evaluate the relationship between AIs and the risk of RA, but compared inflammatory signs in symptomatic patients. Therefore, whether the exposure to AI may induce inflammatory arthritis or RA is still an open question. Chen *et al* retrospectively analysed data from 238 880 women with breast cancer in USA, evaluating the relationship between the risk of Systemic Lupus Erythematosus (SLE) or RA and the exposure to selective oestrogen receptor modulators—SERM (tamoxifen, raloxifene and toremifene) or AIs (anastrozole, exemestane, formestane, letrozole and aminoglutethimide).^14^ Compared with the general population, both patients exposed to SERM and AIs showed an increased risk of RA, with the highest risk in patients with persistent (>12 months) exposure to SERMs; concerning the risk of developing SLE, instead, only SERM were associated with a significantly higher risk. These findings are supported by biological data, as stated by a recent review by Alpizar-Rodriguez *et al*^15^; oestrogens, in fact, are supposed to act as either anti-inflammatory or proinflammatory agents, depending on serum concentrations, ovarian ageing, distribution of oestrogen receptors (ER). An acute decline in oestrogens bioavailability is thought to be responsible of the proinflammatory effect resulting in increased overall risk of RA development (postmenopausal period, early menopausal age, postpartum, use of oestrogens inhibiting drugs) and is already known that inflammatory mediators are thereby responsible for aromatase activation, conversely resulting in increased conversion of androgens to oestrogens.^16^

On this basis, the purpose of this study was to evaluate the risk of developing RA in a large population-based sample of women with breast cancer treated with AIs after mastectomy.

## Methods

This is a nested retrospective cohort study on administrative healthcare databases (AHD) of Lombardy Region, Italy (>10 000 000 inhabitants).

Data were retrieved between 1 January 2004 and 31 December 2013 by record linkage and include demographic variables (birth date, gender, death date), drug delivery (Anatomical Therapeutic Chemical (ATC) classification, date of delivery, quantity), disease certification by rheumatologist (‘exemption code’, date of certification), outpatient services (International Classification of Diseases (ICD)-9 CM procedure code and date) and hospital admissions (beginning and end of hospitalisation, ICD-9-CM diagnosis code and disease-related group (DRG) classification).

The database population included patients with RA (prevalent and incident cases) and four age-matched and sex-matched controls from the general population. The study population included the subgroup of women who underwent to mastectomy (DRG 257-258-259-260 or ICD-9-CM diagnosis 174), with at least one exposure to adjuvant endocrine therapy in the follow-up, and without already established diagnosis of RA at the time of mastectomy. Incidence of RA occurring during the follow-up was defined according to the first step of the RECord linkage on Rheumatic Diseases (RECORD) study algorithm: certification of RA by a rheumatologist (exemption code 006.714.0) or RA code (714.0) in the hospital discharge form or the prescription of leflunomide, tocilizumab, abatacept or gold salts.^17^

Access to data was granted by the General Directorate of Health for the purpose of RECORD study protocol of analysis, a study promoted by the Italian Society for Rheumatology which aims to set up a national surveillance system to monitor the health burden of rheumatic diseases in Italy using data from AHD; aims of this project are evaluating the frequency of RA burden, the impact of the disease and its treatment on disease outcomes at population level and the quality of care delivered to patients with RA.

Level of neoplasia was defined according to relevant (ICD-9-CM codes as localised (ICD9-CM 174.0–174.9), node-positive (ICD9-CM 196.3), metastatic (ICD9-CM 198.81), unspecified.

Exposures to tamoxifen, anastrozole, letrozole or exemestane (ATC L02BA01, L02BG03 and L02BG04 L02BG06) were defined by the drug delivery recorded in the AHD. Exposure was considered changing during the follow-up. A patient was considered exposed to a specific treatment from the first prescription of drug until the last one plus 6 months, to consider the coverage period of drug also after its withdrawal, or until the first prescription of subsequent drug, and censored accordingly.

### Statistical methods

The association between AIs exposure and RA was assessed by survival models of RA development using death as competing risk, with time-dependent covariates. Results were presented as HR and 95% CI, crude and adjusted for prespecified confounders (age at surgery, level of neoplasia). Interaction with age and time varying effects were also explored. The analyses were performed using the R statistical environment.^18^

## Results

A total of 7533 women with breast cancer were eligible to be included in the analyses. Demographic and specific ICD-9-CM diagnoses are reported in table 1.

**Table 1:** Study sample characteristics

N	7533
Age (years), median, SD (IQR)	65.21, 11.72 (58–74)
ICD9-CM code, N (%)	
*174.0 Nipple and areola*	97 (1.29)
*174.1 Central portion*	407 (5.40)
*174.2 Upper-inner quadrant*	690 (9.16)
*174.3 Lower-inner quadrant*	437 (5.80)
*174.4 Upper-outer quadrant*	2638 (35.02)
*174.5 Lower-outer quadrant*	490 (6.50)
*174.6 Axillary tail*	19 (0.25)
*174.8 Other specified sites of female breast*	791 (10.50)
*174.9 Malignant neoplasm of breast (female), unspecified*	1765 (23.43)
*196.3 Secondary malignant neoplasm of lymph nodes*	1801 (23.91)
*198.81 Secondary malignant neoplasm of breast*	19 (0.25)

ICD9-CM, international classification of diseases-clinical modification.

The study sample remained under observation for a total of 26 105.9 person-year. In this observation time, a total of 113 new cases of RA developed, corresponding to a crude incident rate (IR) of 4.33 per 1000 person-years (95% CI 3.57 to 5.20).

The number of incident cases of RA, exposure periods and person time according to the exposure to different drugs are detailed in table 2. A total of 1507 women was exposed either to tamoxifen or to AIs; a total of 23 new cases of RA occurred in this subgroup.

**Table 2:** Incidence rate in the study sample according to drug exposure

	Incident rheumatoid arthritis	Person-years	Exposure periods	Incident rate *1000/yr (95% CI)
Tamoxifen	26	8650.2	3371	3.01 (1.96 to 4.40)
Aromatase inhibitors	87	17 455.7	6815	4.98 (3.99 to 6.15)
Anastrozole	50	9457.8	3170	5.29 (3.92 to 6.97)
Letrozole	30	6626.6	2785	4.53 (3.05 to 6.46)
Exemestane	7	1371.3	860	5.10 (2.05 to 10.5)

Using tamoxifen as reference category, AIs therapy was associated with an increased risk of RA (adjusted HR 1.62 (95%CI 1.03 to 2.56)), in particular in patients receiving anastrozole, even after adjusting for age and level of neoplasia (table 3). Interaction with age was not statistically significant and no time varying effects were observed.

**Table 3:** Hazard ratios of development of rheumatoid arthritis according to drug exposure

	Crude HR	Adjusted HR (95% CI)*	Competitive risk adjusted HR (95% CI)*†
Tamoxifen	Reference	Reference	Reference
Aromatase inhibitors	1.67 (1.08 to 2.60)	1.64 (1.04 to 2.58)	1.62 (1.03 to 2.56)
Anastrozole	1.77 (1.10 to 2.85)	1.73 (1.06 to 2.81)	1.75 (1.07 to 2.86)
Letrozole	1.51 (0.89 to 2.56)	1.49 (0.87 to 2.56)	1.47 (0.86 to 2.51)
Exemestane	1.78 (0.76 to 4.17)	1.75 (0.74 to 4.16)	1.47 (0.63 to 3.43)

*Adjusted for age at the beginning of the exposure period, level of neoplasia.

†Competing risk survival model.

## Discussion

In a large population-based sample of women with breast cancer, the exposure to AIs compared with tamoxifen is associated with a 60% increase of the risk of developing RA, independently by the cancer severity and the relationship of age with indication to specific drugs.

Our main results are consistent with those deriving from a large, US population-based observational study on AHD by Chen *et al*.^14^ The analysis of data from 238 880 women with breast cancer in USA between 1999 and 2013 marked a relationship between the probability of RA and the exposure to SERM or AIs. Compared with the general population, patients exposed to SERM and AIs for less than 11 months showed an increased risk of RA (OR for SERM 1.26, 95% CI 1.13 to 1.41; OR for AIs 1.32, 95% CI 1.21 to 1.44), with the highest risk in patients with persistent (>12 moths) exposure to SERMs (OR for SERM 2.41, 95% CI 1.92 to 3.02; OR for AIs 1.85, 95% CI 1.57 to 2.17).

Our study specifically aimed to compare the risk of RA associated to AIs compared with tamoxifen, given the higher occurrence of AIMMS in patients treated with AIs. Our results are also supported by basic science evidence. Oestrogens are produced from androgenic precursors through the enzymatic activity of aromatase and exert wide effects by binding to receptors present on many tissues. Aromatase is the key enzyme for the conversion of androgens into oestrogens and in patients with RA, low levels of androgens and high levels of oestrone are found in the synovial fluid.^16^ A possible but speculative explanation of our result could also be provided by an experimental study on mouse models of human RA, in which the administration of anastrozole significantly increased the severity of arthritis. Anastrozole induced increased levels of proinflammatory cytokines and decreased levels of interleukin (IL)-4, IL-10 secretion, it suppressed the differentiation of naive T cells to T reg cells and blocked the balance of IgG2a/IgG1 in peripheral blood.^19^

This study has several limitations. The identification of women with breast cancer is limited to those undergoing mastectomy during the observation period, possibly leading to a slight selection of our study population. Drug exposure is only indirectly evaluated based on the drug delivery, which could over estimate the true exposure to the drug due to incomplete adherence. However, the attribution of RA cases to the period of drug coverage makes plausible the association between drug and outcome.

Several factors, and age in particular, influence prescription of a specific drug and the likelihood of developing RA. Tamoxifen is currently most commonly indicated in premenopausal women, which have a low incidence of RA. In line with this consideration, after adjusting for age, the HR for AIs decreased, unmasking this possible confounding. Further interaction between age and drugs has been explored. In particular, cancer severity is an important factor that may influence survival and therefore the probability to develop RA. Applying survival models for competing risks, the influence on mortality is controlled in the analyses, although the probability of diagnosing RA in late stages of cancer is reasonably lower than in patients in cancer remission. The attribution of the events to the drug to which the patients was concurrently exposed by covariates helped in controlling this potential confounding. However, carry-over effect is still possible in patients switching for one drug.

The diagnosis of RA is based on administrative variables and prone to high misclassification. In a previous study, we measured misclassification of AHD for the identification of RA at population level,^17^ validating its application in the database we used for these analyses and showing an accuracy consistent with other literature data, allowing this algorithm to be valid (in terms of accuracy) to estimate disease burden. Furthermore, misclassification is not likely to be different in different drug exposure levels. Finally, though this is a population-based study including a large sample size, RA occurrence is a rare event and the study is not adequately powered to detect small effect sizes. For this reason, the observed increase of the risk of RA (about 60%) attributed to AIs should be confirmed in subsequent studies.

Our study strengthens the body of evidence on the AI-induced musculoskeletal symptoms, suggesting that an increased risk of RA might be present in women treated with AIs. A careful examination and strong interaction between oncologists and rheumatologist are recommended to achieve a correct diagnosis and optimise the management of AI-induced musculoskeletal symptoms.
